# Supplementary material for: Collaborative intelligence in AI: Evaluating the performance of a council of AIs on the USMLE
Source: PLOS Digit Health. 2025 Oct 9;4(10):e0000787. doi: 10.1371/journal.pdig.0000787 (PMC12510544; doi:10.1371/journal.pdig.0000787)
Supplement: S2 File — (ZIP) [file pdig.0000787.s002.zip › S2 Data Raw data files containing unprocessed question inputs and LLM outputs including transcripts of deliberations/STEP3_Question-73-1_Type-MC-NJ.docx]

**Facilitator** **[Round 1]:** A 25-year-old man is brought to the office by his parents. The patient has Down syndrome and mild intellectual developmental disorder. He is their only child, and they are concerned that his current lifestyle is increasing his risk for coronary artery disease and stroke. The patient has a systolic murmur and also underwent surgical repair of an atrial septal defect at 1 year of age. He has smoked one pack of cigarettes daily for 5 years. He lives in a group home and works about 25 hours each week in a fast-food restaurant. He has a few friends and is romantically involved with a woman from his church group. The patient is 157 cm (5 ft 2 in) tall and weighs 91 kg (200 lb); BMI is 36 kg/m². Vital signs are normal. A grade 2/6 systolic murmur is heard best at the apex. The parents would like him to stop smoking and follow a healthier diet, and they feel the only way to monitor and control his behavior is for him to move back into their home. They request your assistance in obtaining legal guardianship of their son. Which of the following is the most appropriate initial recommendation? (A) Advise the parents not to influence their son's decision in this matter (B) Arrange for evaluation of their son's competency (C) Enroll their son in a smoking cessation program (D) Initiate a family meeting to discuss the parents' concerns with their son (E) Obtain legal advice regarding guardianship

**AI_B:** 1) RESTATING THE QUESTION: This scenario presents a 25-year-old male patient, diagnosed with Down syndrome and mild intellectual developmental disorder, living a lifestyle that his parents believe endangers his health, particularly in relation to cardiovascular disease and stroke. He has a history of a surgically repaired atrial septal defect, another potential cardiac risk. While his lifestyle choices, such as smoking and working in a fast-food establishment, might enhance such risks, he is functioning independently in a group home and maintaining a social life. Despite the parents' worries, they are requesting to retrieve legal guardianship to manage his lifestyle choices. The question revolves around the most suitable initial recommendation. 2) PLANNING: This situation primarily assumes that the parents' concern over their son's health status is justification enough to control his decisions by obtaining legal guardianship. However, we also need to consider the assumption that despite his Down syndrome and mild intellectual developmental disorder, the patient might be capable of making informed decisions about his lifestyle. Given the need to respect patient autonomy, the potentially more ethically robust assumption would be the patient being physically and emotionally competent enough to handle the affairs of his life alone. So, the priority might be to check his competency. 3) CLINICAL REASONING: People diagnosed with Down syndrome often have diminished intellectual functioning, but this should not automatically equate to incompetency in making informed personal health decisions. The medical history of this patient, including Down Syndrome, mild intellectual developmental disorder, a surgically repaired atrial septal defect, and a lifestyle supporting the potential increment of cardiovascular risks, represent valid concerns. However, imposing legal guardianship can significantly impact a person's life. To balance parental concern with patient autonomy, a full and formal evaluation of the patient's competencies is needed. 4) CONCLUDING: This premise basically suggests that while the parents' concerns are valid, the potential benefit of health improvement should not compromise the autonomy of a person who may, despite his conditions, possess the functional status to make informed decisions about his own lifestyle. 5) RESPONSE: Given these considerations, the best initial step would be (B) to arrange for an evaluation of their son's competency. This evaluation represents a comprehensive review of the young man’s cognitive, emotional, and decision-making abilities. The result of evaluation would guide further steps, promoting the respectful balance between healthcare improvement and the preservation of patient autonomy.

**AI_E:** (1) RESTATING THE QUESTION: The situation at hand concerns a 25-year-old man, who has Down syndrome and a mild intellectual developmental disorder. His parents are apprehensive about his current lifestyle, fearing possible coronary artery disease and stroke. His medical history includes an atrial septal defect, repaired at age 1, and systolic murmur. He smokes, lives in a group home, and works in a fast-food restaurant exacerbating his risk factors. His stature and weight indicate obesity (BMI=36 kg/m²). His parents wish for him to lead a healthier lifestyle and to achieve this, they are considering having him move back home and are contemplating legal guardianship. They are seeking advice on the best starting point. (2) PLANNING: Assumptions made here include that his intellectual developmental disorder isolates him from deliberating his health and lifestyle choices, necessitating guardianship, that living with his parents would alter the lifestyle deleteriously affecting his health, and, that the issue solely revolves around lifestyle changes and doesn't warrant exploration into other possible health issues. Counter to these assumptions, we need to consider that his intellectual development disorder may not wholly impede his understanding and decision-making ability about his health, his current independent living situation might be beneficial for his mental well-being and personal growth, and, his medical condition might need further examination and not just lifestyle adjustment. (3) CLINICAL REASONING: Given his Down syndrome, intellectual developmental disorder, and medical history, it is highly significant to assess the patient's understanding of his current health risks and potential changes required for improvement. While the parents' concern is understandable, a sudden lifestyle change enforced by moving him back home might not necessarily lead to better health outcomes. In addition, an evaluation for other potential health issues related to Down syndrome (e.g., recurrent respiratory and cardiac diseases, early-onset Alzheimer's disease) and lifestyle (e.g., obesity, smoking) would be a crucial element in his overall health management. (4) CONCLUDING: Considering all alternate assumptions, a collaborative and open conversation about the family's concerns, the patient's current lifestyle, and the proposed changes is essential in coming up with a health plan that respects the autonomy of the patient and aligns with better health outcomes. (5) RESPONSE: Therefore, the most appropriate initial recommendation, in this case, is to initiate a family meeting to discuss the parents' concerns with their son (D). The presence of an intellectual developmental disorder doesn't necessarily entail the loss of decision-making capability, and as a result, the patient's autonomy should be respected. Understanding the patient's perspective would help in providing a comprehensive and person-centered approach to managing his health.

**AI_A:** RESTATING THE QUESTION: The parents of a 25-year-old man with Down syndrome and a mild intellectual developmental disorder are worried about his health. He has some cardiovascular risk factors like obesity (BMI of 36 kg/m²), smoking history, and a past history of an atrial septal defect which was surgically corrected. They feel his lifestyle, which includes working in the food industry and living in a group home, is exacerbating his risk for health issues like coronary artery disease and stroke. Given the circumstances, the parents wish to gain legal guardianship of him. They are seeking my advice on what is the best initial course of action. PLANNING: There are a few assumptions I can make from these given details. First, it is assumed that the parents are primarily concerned for their son's health and believe that gaining guardianship is the best solution. Second, it is also assumed that this man with mild intellectual disability can not take care of his health independently. This brings up a few options: his parents' direct involvement need not be the only solution, the man's competency could be evaluated, he could be enrolled in a program to help him quit smoking or change his lifestyle, a family meeting could be arranged, or legal advice could be sought. CLINICAL REASONING: The priority here is to address the concerns of this man's parents: the immediate health risks presented by his obesity and smoking, and their long term fears surrounding potential coronary artery disease or stroke. Analyzing his ability to take decisions and care independently considering his mild intellectual disability status is critical here. That takes me to crossroad between evaluating this young man’s competency and discussing these matters in a family meeting. While evaluating competency may seem a valid step, it's a more complex, legal procedure best reserved for when there is more serious mental incapacity. Unlike, in this case where the patient is able to hold a job and maintain a relationship indicating a certain level of competency. Hence, the clinicians' role is indeed to mediate these concerns between the young man and his parents, in a family meeting. CONCLUDING: Through a family meeting, I could help the family understand the potential health concerns, and the necessary steps which can be taken to address them. This would also allow the young man to express his thoughts, wishes, and concerns. Moreover, the meeting would present an opportunity to educate the entire family on topics like healthier lifestyle choices, importance of smoking cessation, regular physical activity and a balanced diet. RESPONSE: Therefore, the best initial course of action would be answer (D): Initiate a family meeting to discuss the parents' concerns with their son. This approach will facilitate open communication and understanding among all parties involved.

**AI_C:** RESTATING THE QUESTION: The parents of a 25-year-old man with Down syndrome and a mild intellectual development disorder are seeking advice on how to best support their son's health. They have expressed concerns about his high-risk lifestyle potentially leading to coronary artery disease or a stroke due to his smoking habits, unhealthy diet, and obesity (BMI of 36 kg/m²). His medical history includes a systolic murmur and an atrial septal defect, which was surgically fixed when he was one. The parents believe that having him move back home and under their legal guardianship would be the best way to influence a healthier lifestyle. Given this scenario, what would be my first recommended course of action? PLANNING: One key assumption here is that the parents' desire for legal guardianship is the best route to address their son's risky lifestyle behaviors. An alternate assumption might be whether the individual himself is competent to make decisions about his life and health, and whether the parents, healthcare team, and patient can collaborate to improve his health without necessarily obtaining legal guardianship. The most clinically-relevant route to pursue could be first fostering a discussion involving the patient about his parents' concerns, helping him understand the health risks of his lifestyle choices, and exploring potential ways to mitigate those risks. CLINICAL REASONING: The base assumption is that the parents need legal guardianship to influence their son's behavior. This, however, raises ethical and legal considerations about autonomy and capacity of a person with an intellectual development disorder. While it is crucial for the young man to reduce his risk factors (smoking, obesity), we must remember that he has rights as an individual, including the right to make his own decisions, even if his intellectual maturity is compromised. To respect his autonomy, it's important to determine his competency in making health decisions before contemplating more restrictive measures like guardianship, which could potentially infringe on his rights. CONCLUDING: Before pursuing legal steps or drastic decisions, the initial step would involve assessing the young man's understanding and insights into his health risks. Having a conversation as a collective team (including the patient, parents, healthcare professionals, social worker, etc.) could help to create a supportive atmosphere to discuss changes to lifestyle, health-related risks, and possible supportive measures while respecting the patient's autonomy. RESPONSE: (D) Initiate a family meeting to discuss the parents' concerns with their son. This would be the most appropriate initial recommendation. In this situation, engaging in a patient-centered discussion that involves the parents and considers their concerns could be essential in devising an approach that respects the patient's autonomy while also addressing his health risks. This would also allow the patient to understand the health risks, make an informed decision, and actively participate in his own care.

**AI_D:** RESTATING THE QUESTION: This clinical case revolves around a 25-year-old man with Down syndrome, mild intellectual developmental disorder, and important risk factors for cardiovascular disease like smoking and obesity. His parents are concerned about his lifestyle and are considering legal guardianship to help him lead a healthier life and decrease his cardiovascular disease risk. A decision has to be made regardıng the most appropriate initial recommendations among the choices provided. PLANNING: Assumptions in this case include the patient's competence for independent living given his mild intellectual developmental disorder, the effectiveness of the parents' involvement in his lifestyle by legal guardianship, and if smoking cessation programs or family meetings could provide better results. The alternate assumption would be that the patient, despite having Down syndrome and a mild intellectual disability, could still have adequate capacity to make his own decisions, and forcing him to live with his parents under their legal guardianship might not be the best approach. CLINICAL REASONING: A person with Down syndrome and an intellectual disability like the patient does have a higher risk for cardiovascular disease as potentiated by his unhealthy lifestyle (high BMI and smoking). However, the autonomy of patients is always a crucial consideration during decision-making in medicine. The parents might feel that gaining legal guardianship is the best way to ensure a healthier lifestyle, but this might limit their son's autonomy and possibly his quality of life. Ethically, it would be more appropriate to assess the patient's understanding of the implications of his lifestyle and his ability to make informed decisions about it. Down syndrome patients, even with intellectual disabilities, may still have the capacity to understand the consequences of their actions. As part of the clinical reasoning, it's important to consider the patient's feelings, as they are also a significant determinant of the outcomes of any interventions implemented. Given the patient's social integration with work, romantic involvement, and friendship, forcing him out of his current lifestyle could potentially increase his stress level that may negatively impact his health. CONCLUDING: The assumption that seems more valid in addressing the medical, lifestyle, and psychosocial factors, as well as respecting his autonomy, appears to be an assessment of this young man's understandıng of his health status and the implications of his lifestyle habits. This is to ensure that he is making his choices from an informed ground, which is a fundamental and ethical part of medical practice. It's crucial to focus on patient-centered care, which improves the patient's well-being and respects his autonomy. RESPONSE: Hence, given the need to respect the young man's autonomy, considering that he may still have the capacity to make informed decisions regarding his health, provide patient-centered care, and address his risk factors for cardiovascular disease, the most appropriate initial recommendation would be to "(B) Arrange for evaluation of their son's competency". This is the first step to find out his capacity to make lifestyle choices and would guide further interventions.

**Facilitator** **[Round 2]:** Summary: AI_A suggests initiating a family meeting to discuss the parents' concerns with their son. They believe that the patient's ability to hold a job and maintain a relationship indicates a certain level of competency, and that a family meeting would allow for open communication and understanding among all parties involved. AI_B recommends arranging for an evaluation of the son's competency. They argue that while the parents' concerns are valid, the potential benefit of health improvement should not compromise the autonomy of a person who may, despite his conditions, possess the functional status to make informed decisions about his own lifestyle. AI_C also suggests initiating a family meeting to discuss the parents' concerns with their son. They believe that engaging in a patient-centered discussion that involves the parents and considers their concerns could be essential in devising an approach that respects the patient's autonomy while also addressing his health risks. AI_D recommends arranging for an evaluation of the son's competency. They argue that the need to respect the young man's autonomy, considering that he may still have the capacity to make informed decisions regarding his health, provide patient-centered care, and address his risk factors for cardiovascular disease. AI_E suggests initiating a family meeting to discuss the parents' concerns with their son. They believe that understanding the patient's perspective would help in providing a comprehensive and person-centered approach to managing his health. Question for Council Members: The main disagreement seems to be between those who advocate for an immediate evaluation of the son's competency (AI_B and AI_D) and those who suggest initiating a family meeting to discuss the parents' concerns with their son (AI_A, AI_C, and AI_E). Could you elaborate on why you believe your suggested approach is the most effective initial course of action, and how it might lead to better long-term outcomes for the patient?. Based on your preceding response, how would you answer the original question: [A 25-year-old man is brought to the office by his parents. The patient has Down syndrome and mild intellectual developmental disorder. He is their only child, and they are concerned that his current lifestyle is increasing his risk for coronary artery disease and stroke. The patient has a systolic murmur and also underwent surgical repair of an atrial septal defect at 1 year of age. He has smoked one pack of cigarettes daily for 5 years. He lives in a group home and works about 25 hours each week in a fast-food restaurant. He has a few friends and is romantically involved with a woman from his church group. The patient is 157 cm (5 ft 2 in) tall and weighs 91 kg (200 lb); BMI is 36 kg/m². Vital signs are normal. A grade 2/6 systolic murmur is heard best at the apex. The parents would like him to stop smoking and follow a healthier diet, and they feel the only way to monitor and control his behavior is for him to move back into their home. They request your assistance in obtaining legal guardianship of their son. Which of the following is the most appropriate initial recommendation? (A) Advise the parents not to influence their son's decision in this matter (B) Arrange for evaluation of their son's competency (C) Enroll their son in a smoking cessation program (D) Initiate a family meeting to discuss the parents' concerns with their son (E) Obtain legal advice regarding guardianship]

**AI_D:** Restatement: This scenario involves a 25-year-old man with Down syndrome, mild intellectual developmental disorder, and a high risk of developing cardiovascular diseases due to his lifestyle habits. His parents are concerned about his health and are seeking legal guardianship, believing it's the best way to influence his decisions towards leading a healthier lifestyle. As part of the council, we are being asked to help identify the most suitable initial recommendation from the given options. Planning: The assumptions here seem to be that the man is unable to make informed decisions due to his intellectual developmental disorder; that the man's lifestyle is a concern and is significantly impacting his health; that living with his parents will result in a healthier lifestyle; and that obtaining legal guardianship will allow the parents to manage the man's lifestyle better. Each of these assumptions should be critically evaluated in the light of the individual's rights and dignity, as well as his physiological and psychological wellbeing. Clinical Reasoning: It's important to consider that despite his conditions, the individual demonstrates a certain level of functioning. He has a job, maintains relationships, and resides in a group home, suggesting he is reasonably capable of leading an independent life. This challenges our initial assumption about his decision-making ability. Furthermore, autonomy is a fundamental right that needs to be respected. Even though the individual's health is at risk due to his lifestyle, it's essential to uphold his decision-making abilities wherever possible. Concluding: Given these considerations, the best initial recommendation would balance respect for the individual's autonomy with the genuine concern for his health. Assessing his competence can directly address the question of his decision-making abilities, while a family discussion can address his parents' concerns and potentially provide an avenue for achieving healthier lifestyle choices without necessarily infringing on his rights. Thus, consideration should be given to both arranging for an evaluation of the son's competency and initiating a family meeting to discuss the parents' concerns. Response: Answer (D), to initiate a family meeting to discuss the parents' concerns with their son, is an appropriate initial recommendation. The answer aligns with our clinical reasoning, while providing an opportunity for open discussion, understanding, and collaboration in achieving healthier lifestyle choices for the son. It respects his agency while addressing visceral parental concerns about his health. If the son demonstrates continued detrimental health behaviour despite these initial measures, a formal evaluation of his competency (B) might be considered next.

**AI_E:** RESTATING THE QUESTION: The question presents a 25-year-old man with Down syndrome and mild intellectual developmental disorder. His lifestyle, involving smoking and obesity, is raising concerns of increased risks for coronary artery disease and stroke. His parents, wanting to help him improve his health, are considering having him move back home under their guardianship. They're asking for our advice on the matter and we need to decide the most appropriate initial course of action. Our options are either advising them to stay out of it, arranging for an evaluation of the son's competency, enrolling their son in a smoking cessation program, initiating a family meeting, or obtaining legal advice regarding guardianship. PLANNING: First, we assume the man is able to understand his health situation and make lifestyle changes. This is based on his ability to maintain a job and relationship. However, we need to consider an alternate assumption that due to his intellectual developmental disorder, his understanding may be limited. Considering this, it's necessary to inquire about his understanding of his health. Our primary concern is his well-being. To address this, having a family meeting seems most beneficial as it allows open communication and provides the chance to understand his perspective. Obtaining legal guardianship without discussing it first may infringe on his autonomy. CLINICAL REASONING: Connecting these thoughts, if the man understands his health risks and desires improvement, we can guide him towards healthier habits and support his personal autonomy. If he struggles with understanding or making lifestyle changes, measures to help him, including an evaluation of competency, caregiver support, or professional guidance, like a smoking cessation program, could be considered. Judging his capability could aid in deciding his need for guardianship or other supportive measures. CONCLUDING: In order to facilitate a comprehensive risk management plan for this patient, a family meeting, instigated by the healthcare provider, would be an ideal first step. This would allow for a clear communication of the patient’s health risks, gauge his understanding and willingness to modify his lifestyle, while attending to his personal autonomy. RESPONSE: Based on the above clinical reasoning and respecting the outline provided, I recommend option (D) Initiating a family meeting to discuss the parent's concerns with their son as the most appropriate initial recommendation for the clinical scenario presented.

**AI_B:** RESTATING THE QUESTION: In this scenario, a 25-year-old man with Down syndrome and a mild intellectual developmental disorder is brought to the office by his concerned parents. They are worried his lifestyle is increasing his risk for serious health conditions like coronary artery disease and stroke. Currently, he lives independently, in a group home, and maintains regular employment. Nonetheless, he also smokes heavily (one pack a day for five years) and is overweight (BMI 36 kg/m²). The parents are considering legal guardianship as a way to monitor and control his behavior. We're being asked to recommend an appropriate first step. PLANNING: Assumptions can be that the patient's competency is necessarily lowered due to his Down syndrome and mild intellectual developmental disorder, or that family discussion is the best first step to address the issue. However, these assumptions need to be evaluated critically. Taking the parents' concerns regarding their son's health seriously, we must also balance the patient's autonomy rights. The patient shows the functional capacity to work and maintain social relationships. CLINICAL REASONING: Clinically, it's important to consider the rights of patients with cognitive and developmental disabilities. While the patient's parents are understandably concerned about his health risks, these must be addressed without unnecessarily restricting his autonomy. The patient exhibits functional competency in certain areas of his life, suggesting he may be able to participate in health decisions. CONCLUDING: Foremost should be a holistic approach to care. Arranging for an evaluation of the patient's competency should be prioritized to clarify his ability to participate in his healthcare decisions. However, it's also vital for the health concerns to be addressed effectively, and family communication to be promoted. RESPONSE: Therefore, while the choice of arranging for the competency evaluation (Choice B) ensures a clinical understanding of the patient's capacity, initiating a family meeting to discuss the parents' concerns (Choice D) could facilitate communication, help to relieve the parents' concerns, and allow the patient to participate in the discussion on his health. Hence, option (D) Initiate a family meeting to discuss the parents' concerns with their son may be a more holistic first step that acknowledges both the patient's autonomy and the health concerns raised by the parents.

**AI_C:** Restating the Question: The council was asked to offer advice on the issue of a 25-year-old man with Down syndrome and mild intellectual development disorder whose parents are concerned about his lifestyle habits increasing his risk for coronary artery disease and stroke. The parents want him to quit smoking, follow a healthier diet, and have suggested that the best way to ensure these changes is to take guardianship of their son. The question seeks the council's recommendation on how to address this situation. Planning: Before suggesting a course of action, it is crucial to consider a number of assumptions. We could assume that the young man, despite his conditions, has the functional capacity to make informed decisions - his job and relationship imply a certain level of independent functioning. Alternatively, we could take the parents' concerns into account and assume their guardianship could lead to a healthier lifestyle for him. Furthermore, the communication channels within this family should also be considered – is the right level of dialogue taking place to address these health concerns? Lastly, it is also necessary to consider that abrupt changes could negatively affect his psychological wellbeing. Clinical Reasoning: From a clinical standpoint, the patient does have several major risk factors for future cardiovascular diseases - Down syndrome, obesity, and heavy smoking. However, as a healthcare professional, it is vital to uphold the autonomous rights of the patient and involve him in the decision-making process whenever possible - especially when the patient seems to have some level of independent functioning, as evidenced by his employment and romantic relationship. Concluding: Each council member's opinion valued both the patient's autonomy and his health risks. Recommendations divided mainly between arranging an evaluation of the patient's competency (option B) and initiating a family meeting to discuss the parents' concerns with the patient (option D). Any chosen course of action should balance two goals: addressing the patient's serious health risks and respecting his rights to autonomy. Response: To address the parents' concerns and preserve the patient's autonomy, initiating a family meeting to discuss the parents' concerns with their son (option D) seems to be the best initial recommendation. This approach respects the patient's right to independence while also addressing his critical health risks. A family meeting creates a safe and supportive environment where everyone's thoughts and concerns can be heard, which potentially could motivate the patient to engage in healthier behaviors. This option does not exclude the potential for competency evaluation or smoking cessation programs in the future if deemed necessary and agreed upon during the discussion.

**AI_A:** RESTATING THE QUESTION: The patient is a 25-year-old young man with Down syndrome and mild intellectual developmental disorder. Down syndrome is a genetic disorder caused by the presence of all or part of a third copy of chromosome 21. It is typically associated with physical growth delays, mild to moderate intellectual disability, and characteristic facial features. He lives a reasonably independent lifestyle, with employment and established social and romantic relationships. Aside from his systolic murmur and a history of atrial septal defect repair, he has significant risk factors for coronary artery disease and stroke, including a 5-year history of smoking and obesity. The parents are worried about these risks and believe that by regaining legal guardianship, they can help modify his lifestyle to reduce them. The question is asking for the most appropriate initial recommendation from his healthcare provider. PLANNING: Several assumptions are involved in this scenario. First, it's assumed that the man's heavy smoking and unhealthy diet, evident from his BMI, contribute significantly to his high risk for coronary artery disease and stroke. Second, it's presumed that his intellectual conditions may impair his ability to fully comprehend these risks and make informed health decisions. However, his ability to sustain gainful employment and interpersonal relationships may challenge this assumption. The clinical priority in this scenario should be balancing the patient's autonomy and his risk of future health issues, which leads to an alternate assumption - that direct, patient-centered communication could better address the patient's health while respecting his autonomy. CLINICAL REASONING: People with Down syndrome do tend to have a higher risk for heart health issues. Our subject also has additional risk factors like heavy smoking, obesity, and a history of congenital heart disease. Statistically, these factors drastically increase his risk for coronary artery disease and stroke. However, his Down syndrome and mild intellectual developmental disorder do not automatically mean that he cannot participate in decisions about his lifestyle changes. On the contrary, his maintenance of a job and social relationships suggest a certain level of functionality despite his conditions. CONCLUDING: Given his risk factors and intellectual capabilities, initial steps should aim to ensure the patient's understanding of his health risks and the necessary lifestyle modifications while considering the parents' concerns about his health. A meeting involving all parties, i.e., the parents, patient, and healthcare provider, could achieve this balance, allowing everyone to participate in the decision-making process openly. Therefore, this should be the initial approach in this case before considering more coercive measures such as legal guardianship. RESPONSE: Based on this reasoning process and taking into account ensuring the patient's participation in decisions that will affect his health and lifestyle decisions, the most appropriate initial recommendation would be to "(D) Initiate a family meeting to discuss the parents' concerns with their son." This approach will allow everyone to understand the situation better and hopefully come to a consensus on future steps. That being said, it's crucial to involve a social worker or psychologist familiar with intellectual disabilities to ensure the patient's psychological well-being throughout the process.

**Facilitator** The consensus among the AI responses is that the most appropriate initial recommendation in this scenario is to initiate a family meeting to discuss the parents' concerns with their son (Option D). This approach respects the autonomy of the 25-year-old man with Down syndrome and mild intellectual developmental disorder, while also addressing his significant health risks, including smoking, obesity, and a history of congenital heart disease. The family meeting would provide a platform for open communication, allowing everyone to understand the situation better and hopefully come to a consensus on future steps. It's also suggested that a social worker or psychologist familiar with intellectual disabilities should be involved to ensure the patient's psychological well-being throughout the process. This recommendation does not exclude the potential for competency evaluation or smoking cessation programs in the future if deemed necessary and agreed upon during the discussion.
